# Evaluation of Systemic Inflammation in Children with Untreated Dental Caries

**DOI:** 10.3290/j.ohpd.c_2122

**Published:** 2025-07-08

**Authors:** Fatma Saraç, / Taymour Abuawwad, / Sinem Öztürk, / Şeyda Korkmaz, / Merve Kaya Saraçgil, / Periş Çelikel, / Sera Şimşek Derelioğlu

**Affiliations:** a Fatma Saraç Pediatric Dentist, Department of Pediatric Dentistry, Atatürk University, Faculty of Dentistry, Erzurum, Turkey. Conceptualisation, methodology, prepared original draft, reviewed and edited manuscript.; b Taymour Abuawwad Pediatric Dentist, Department of Pediatric Dentistry, Atatürk University, Faculty of Dentistry, Erzurum, Turkey. Methodology, data curation, statistically analysis.; c Sinem Öztürk Pediatric Dentist, Department of Pediatric Dentistry, Atatürk University, Faculty of Dentistry, Erzurum, Turkey. Investigation, data curation, formal analysis.; d Şeyda Korkmaz Pediatrician, Department of Pediatrics, Atatürk University, Faculty of Dentistry, Erzurum, Turkey. Methodology, investigation, sample collection.; e Merve Kaya Saraçgil Pediatrician, Department of Pediatrics, Atatürk University, Faculty of Dentistry, Erzurum, Turkey. Methodology, investigation, sample collection.; f Periş Çelikel Pediatric Dentist, Department of Pediatric Dentistry, Atatürk University, Faculty of Dentistry, Erzurum, Turkey. Methodology, investigation, reviewed and edited manuscript.; g Sera Şimşek Derelioğlu Professor, Pediatric Dentist, Department of Pediatric Dentistry, Atatürk Atatürk University, Faculty of Dentistry, Erzurum, Turkey. Study design, methodology, conducted and supervised the study.

**Keywords:** children, dental caries, NLR, PLR, SII, systemic inflammation.

## Abstract

**Purpose:**

To analyse the relation between dmft/DMFT and pufa/PUFA indices and markers of systemic inflammation, such as SII, NLR, PLR NEUT, LYMPH and PLT.

**Materials and Methods:**

The study sample consisted of 59 children with untreated dental caries (patient group) and 59 healthy children without caries (control group). Complete blood count (CBC) parameters were collected from both groups, and systemic inflammation markers, such as the Systemic Immune-Inflammation Index (SII), Neutrophil-to-Lymphocyte Ratio (NLR), and Platelet-to-Lymphocyte Ratio (PLR), were calculated. Additionally, the study utilised the decayed, missing and filled teeth (dmft/DMFT) and pulpal involvement, ulceration, fistula, abscess (pufa/PUFA) indices to assess the severity of caries and pulp disease. Normality was checked using the Shapiro-Wilk test. For non-normally distributed data, the Mann-Whitney U-test was used to compare two independent groups. Pearson’s chi-squared test analysed relationships between categorical variables when expected values exceeded 5. Spearman correlation was applied for continuous variables without normal distribution. All statistical analyses were performed using IBM SPSS 25.

**Results:**

The results demonstrated statistically significant differences in systemic inflammation markers between the two groups. Specifically, children with untreated caries showed statistically significantly higher levels of neutrophils (NEU%), SII, NLR, and PLR compared to the healthy control group. (p < 0.01). Furthermore, a statistically significant decrease in lymphocyte count (LYMPH#) was observed in the patient group compared to the control group. However, no statistically significant correlations were found between the clinical severity of caries (as measured by the dmft/DMFT and pufa/PUFA indices) and systemic inflammation markers.

**Conclusion:**

The results of our study indicated that the inflammatory parameters, including NEU%, NLR, PLR, and SII, were elevated in children with untreated caries compared to those without caries. It has been observed that oral health may affect systemic health in children, thus reconfirming the importance of maintaining good oral and dental health.

Oral health is a crucial adjunct to overall health and a significant driver of quality of life. If neglected, caries can result in numerous adverse outcomes, including infection, discomfort, premature tooth loss, malnutrition, weight reduction, sleep disturbances, speech impairments, psychological and economic issues, and diminished quality of life.^1,3,8,22,30^ Untreated caries allows cariogenic bacteria to invade the pulp tissue, resulting in periapical periodontitis, which may extend to surrounding bone and soft tissues, potentially leading to more severe systemic problems.^12,28^ The systemic effects of untreated caries and the mechanical role of the oral microbial-inflammatory process in these relationships require further investigation through human and animal studies.^28^ The potential for a caries-fostering oral microbiome to spread into systemic circulation is plausible and parallels mechanisms previously studied in periodontal disease. In particular, the involvement of the root canal space or the marginal periodontium is considered the most likely pathway for the direct systemic extension of oral microorganisms.^11^ Host factors and pathogenic properties within the oral microbiota can promote the development of caries and increase the likelihood of systemic dissemination.^12^

Field studies have been carried out around the globe to determine the caries status in different populations. These studies utilise prevalence and incidence rates, along with indices such as DMFT/dmft (decayed, missing, filled teeth).^29,38^ The DMFT/dmft index indicates the current oral health status of patients; however, it inadequately discloses untreated caries-related pulp involvement and the existence of disorders such as dental abscesses, fistulas, and ulcerations. In 2010, Monse et al^21^ created the PUFA/pufa index to obviate the shortcomings of previous indicators. The PUFA index clinically assesses oral diseases stemming from untreated caries and documents pulpal conditions related to the pulp, including caries, ulcerations, fistulas, and abscesses.^13^

The parameters of a complete blood count (CBC) encompass major indicators for assessing systemic inflammation. The interrelations among these characteristics strongly impact therapeutic practice. Studies reveal the interaction of these factors with inflammation and their utility in the diagnosis and prognosis of diverse diseases.^5,17,19^ Furthermore, indices derived from these measures, such as NLR, PLR, and SII, have been applied to assess systemic inflammation and prognosticate various diseases.^15,17,34^

The literature contains few studies analysing the association between untreated caries and related systemic inflammation. In this context, it is crucial to analyse indicators of systemic inflammation in children with elevated dmft/DMFT and pufa/PUFA indices. The present study aimed to analyse the relationship between dmft/DMFT and pufa/PUFA indices and markers of systemic inflammation; specifically, this study investigated the impact of neglected caries and their advanced complications on systemic inflammatory parameters. The findings are intended to contribute to the existing literature regarding the potential systemic effects of untreated caries in children. The null hypothesis is that there is no statistically significant difference in systemic inflammatory markers between children with untreated caries and those without caries.

## MATERIALS AND METHODS 

This prospective cross-sectional comparative study was ethically approved by the Atatürk University Faculty of Medicine Clinical Research Ethics Committee (27.09.2024; No. 540). Additionally, the organisation and acceptance for observational studies during the execution phase, adherence to the “STROBE” guidelines for cross-sectional studies, and consideration of the guidelines prepared for the studies in question were all taken into account.^32^

### Patient Selection

The patient group included a total of 59 patients with oral health parameters of untreated dental caries who underwent dental treatment under general anesthesia at Atatürk University Faculty of Medicine Hospital between 30.09.2024 and 30.12.2024. None of the included patients had any systemic disease.

The control group of our study included a total of 59 children who applied to Atatürk University Faculty of Medicine Hospital Pediatrics outpatient clinic for routine control between 30.09.2024 and 30.12.2024 and did not have dental caries. The ages and sexes of the children in the control group were matched to those in the patient group. Informed consent was obtained from all subjects who participated in the study.

#### Exclusion Criteria

Children with a history of systemic diseases, syndromes, or congenital defects were excluded from our study and control groups. Patients with upper respiratory tract infections and those currently using any medication were excluded from the study during the pediatric clinical examination. Children with caries were excluded from the control group. Additionally, children or parents who declined to participate in the study were excluded from both groups. Patients with anemia or polycythemia, leukopenia or leukocytosis, thrombocytopenia or thrombocytosis in total blood count analysis were excluded from the study.

### Data Collection Method

Oral examinations were performed in all children. Participants receiving dental treatment under general anesthesia usually underwent complete blood count analysis for pre-procedural assessment. Patients in the control group were admitted to the pediatric clinic for normal examinations, and a CBC analysis was also conducted. For each patient, the CBC parameters reported included PLT (PLT#), NEUT (NEUT#), LYMPH (LYMPH#) counts, as well as the percentages of LYMPH (LYMPH%) and NEUT (NEUT%). The subsequent formulas were used for the calculations of NLR, PLR, and SII indices:

SII = (PLT count x NEUT count) ÷ LYMPH count
NLR = NEUT count ÷ LYMPH count
PLR = PLT count ÷ LYMPH count

#### Laboratory analysis

A Sysmex XN-9000 (Sysmex; Kobe, Japan) device was used to quantify neutrophils, lymphocytes, and platelets in whole blood samples contained in EDTA tubes (Vacuette Tube 3 ml K2 EDTA; Greiner Bio-One; Kremsmünster, Austria).

#### Oral examination and calibration

Oral examination was conducted by two pediatric dentists with the help of a registration form. Examiners were calibrated for the dmft and pufa indices. Dental examination was performed under artificial light, using a dental mirror and a periodontal WHO probe in accordance with WHO guidelines.^36^ Caries in children was assessed using the dmft index for primary teeth and the DMFT index for permanent teeth, calculated as the aggregate of decaying teeth (d/D), missing teeth (m/M), and filled teeth (f/F). When assessing extracted teeth, those that had not yet been physiologically replaced by permanent teeth at the relevant age were included in the dmft.

Clinical outcomes of untreated caries were assessed using the PUFA/pufa index. In this index,^21^ “P/p” refers to pulp involvement, such as a tooth with an open pulp chamber or extensive coronal destruction with only the roots remaining. “U/u” represents ulceration caused by trauma from sharp fragments of a dislocated tooth with pulpal involvement or root remnants, which results in mucosal injury to areas such as the tongue or buccal mucosa. “F/f” stands for an active fistula, and “A/a” denotes an abscess. In line with the aim of our study, the total scores of dmft/DMFT, as well as PUFA/pufa, were assessed in individuals with mixed dentition.

### Sample Size Calculation

To calculate the sample size, relevant studies in the literature were reviewed, and an appropriate effect size was determined based on the data reported in these studies. The standardised effect size was calculated as 0.543 based on the findings of Guo et al.^10^ Using this effect size, a significance level of 5% (α = 0.05), and a statistical power of 80% (1-β = 0.80), the minimum required sample size was calculated to be 88 participants in total (44 per group) using G*Power 3.1.9.2 (Heinrich Heine University Düsseldorf, Düsseldorf, Germany ).

### Statistical Analysis

The present study included descriptive statistics (number, percentage, mean, standard deviation, median, minimum and maximum) of the data. The assumption of normal distribution was checked with the Shapiro-Wilk test. When the assumption of normality was violated, the Mann-Whitney U-test was employed to compare two independent groups. Pearson’s chi-quared test was employed to analyse the relationship between categorical variables when the sample size assumption (expected value > 5) was fulfilled. Spearman correlations were used to analyse the relations between continuous measurements that were not normally distributed. Analyses were carried out in IBM SPSS 25 (IBM; Armonk, NY, USA).

## RESULTS

A total of 118 patients were included in the patient (59) and control (59) groups. A statistically significant difference was noted between the ages of the patient and control groups (p<0.05). The age of the patient group was higher than the control group. No statistically significant variation in gender distribution was noted between the two groups (Table 1).

**Table 1:** Age and gender distribution of children in the study by group

	Patient group	Control group	p
Min.–Max.	Mean ± SD (Med)	Min–Max	Mean ± SD (Med)
Age	3–12	5.3 ± 1.9 (5)	2–13	4.3 ± 2.1 (4)	0.001*
Gender	n	%	n	%	p
Female	27	48.2	22	39.3	0.341
Male	29	51.9	34	60.7	
Min-Max: minimum-maximum); Med: median; SD: standard deviation. *p < 0.05, Mann-Whitney U-test, Pearson chi-squared test.

**Fig 1:**
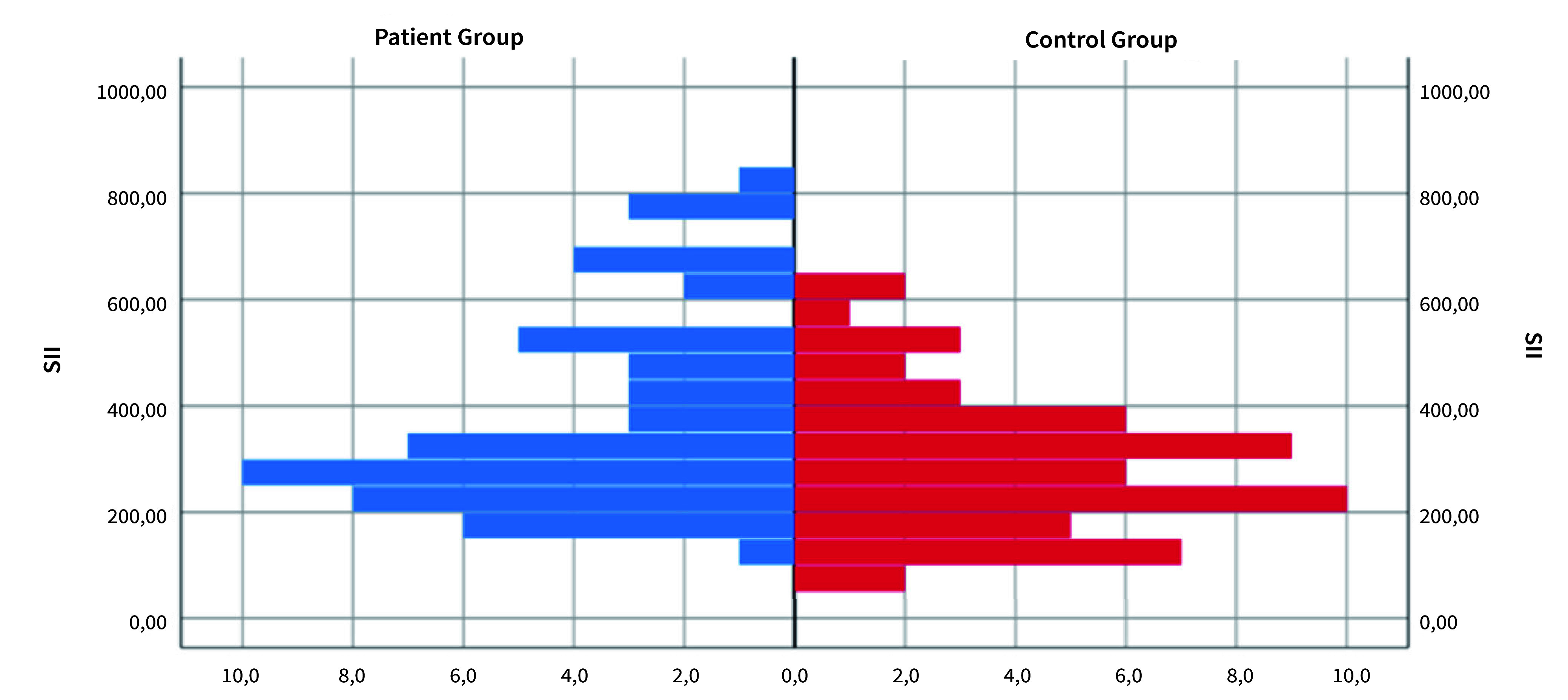
Histogram of frequency distribution of SII measurements based on study groups.

Statistically significant variations were identified between NEU%, LYMPH#, SII, NLR, and PLR measures in the patient and control groups (p<0.05). LYMPH# readings in the control group exceeded those in the patient group. The measures of NEU%, SII, NLR, and PLR in the patient group exceeded those in the healthy group (Table 2, Fig 1.)

**Table 2:** Distribution and comparison of CBC measurements by study group

	Patient group	Control group	p
Min.–Max.	Mean ± SD (Med)	Min–Max	Mean ± SD (Med)
NEUT#	1.8–7.3	3.8 ± 1.4 (3.4)	1.1–7.7	3.5 ± 1.56 (3.4)	0.320
NEU%	26–42.5	51.1 ± 51.7 (43.4)	16.7–59.4	39.4 ± 10.6 (40.5)	0.038*
LYMPH#	2.0–6.4	3.8 ± 0.8 (3.8)	2.0–8.7	4.5 ± 1.5 (4.3)	0.012*
LYMPH%	24.7–68	45.5 ± 9.3 (46.6)	30.8–73.1	50.3 ± 11.0 (48.6)	0.057
PLT#	235–583	375.6 ± 77.9 (379)	258–574	353.7 ± 65.1 (351.5)	0.100
SII	143.9–843.2	384.3 ± 186.6 (320.5)	70.9–641.0	294.5 ± 139.2 (279.0)	0.014*
NLR	0.3–2.1	1.0 ± 0.4 (0.9)	0.2–1.7	0.8 ± 0.3 (0.8)	0.037*
PLR	59.1–156.2	101.3 ± 25.4 (101.2)	41.0–180.1	84.8 ± 26.6 (80.8)	<0.001*
*p<0.05, Mann–Whitney U–test, Min–Max: minimum–maximum); Med: median; SD: standard deviation.

According to Spearman correlation analysis, SII and PUFA/pufa and DMFT/dmft scores in the patient group (Table 3) were not statistically significantly correlated (p > 0.05).

**Table 3:** Correlation analysis between SII and dental caries indices in the patient group

		SII
PUFA/pufa	r	-0.135
	p	0.322
DMFT/dmft	r	-0.145
	p	0.288
r: correlation coefficient, *p<0.05.

## DISCUSSION

The systemic effects of dental caries may manifest through two main mechanisms: first, via infections caused by the translocation of oral microorganisms, and second, through the induction of a pro-inflammatory state.^28^ Many studies have also been conducted on untreated caries, focusing on aspects such as quality of life, growth and development, jaw development, and the psychological effects on children.^1,3,8,22^ However, studies on the effects of untreated caries on systemic inflammation are scarce. Accordingly, we compared the systemic inflammation parameters of children with untreated caries with those of caries-free children. In the present study, the null hypothesis was partially rejected as the LYMPH# measurements in the control group were higher than those in the patient group. Since NEU%, SII, NLR and PLR measurements in the patient group were higher than the healthy group, our null hypothesis was partially accepted.

The inflammatory response is the physiological reaction of the human body against infections. Neutrophils, the first line of defense against bacterial infections, form an essential part of the innate immune system.^16,35^ The platelet count fluctuates based on infectious agents and treatment modalities, and may rise secondarily in reaction to infection or inflammation in some infectious disorders. Platelet counts may not fluctuate significantly in many infectious diseases, as platelets are not the primary mediators of the inflammatory response in the bloodstream.^20,31^ However, viral infections can significantly alter the number and localisation of T and B lymphocytes in the human body.^4,7^

Laboratory indicators of systemic inflammation have gained importance for assessing disease activity and prognosis across various conditions. The literature contains studies on how complete blood count (CBC) parameters interact with inflammation and how they contribute to the diagnosis and prognosis of various diseases.^2,5,17,19^ Studies also exist on the association of CBC parameters with conditions such as odontogenic infections^25^ and periodontitis.^26^ A new generation of biomarkers such as the systemic inflammation index (SII), neutrophil-to-lymphocyte ratio (NLR) and platelet-to-lymphocyte ratio (PLR) have also been utilised in recent years to assess systemic inflammation.^9,25,37^ Lippi et al^18^ concluded that inflammation indices derived from blood cell counts such as NLR and PLR are more sensitive and reliable biomarkers of inflammation than individual parameters of the blood cell line. Given that CBC parameters have limited predictability in precisely assessing infection severity, prognostic indices derived from neutrophil, lymphocyte, and platelet counts are anticipated to yield more robust and dependable outcomes than those based on a solitary element alone.^14^

The present study revealed that the lymphocyte count was within the reference range in both groups, but higher in the healthy group than in the patient group. Lymphocyte percentages were similar in both groups. These results suggested that it may be relatedto physiological lymphocytosis considering the fact that the mean age was lower in the healthy group. The other parameter assessed here was neutrophil count and neutrophil %. Our results indicated that neutrophil % was statistically significantly higher in the untreated caries group than in the caries-free group. Neutrophil count was also higher in the patient group, although not statistically significantly so. This can be elucidated by the bacterial aetiology of caries. The platelet count, another criterion assessed in our study, was within the reference range and was comparable in both groups. Platelet count does not vary much with age; values below 150×10⁹/l are defined as thrombocytopenia and values above 450×10⁹/l as thrombocytosis.^6,33^ Equal platelet count in both groups may be explained by the fact that platelets are not the main inflammatory response agent in bacterial infections.

The present study found that NLR, PLR, and SII measurements were higher in the group with untreated caries, indicating elevated systemic inflammation in these individuals. In a recent study which supports our results, Prikop et al^24^ reported a statistically significant positive correlation between the severity of odontogenic infection and SII. In contrast, our study did not detect a statistically significant correlation between dmft/DMFT and pufa/PUFA scores and the inflammatory markers NLR, PLR and SII. This suggests that while children with untreated caries may exhibit increased systemic inflammatory markers, this elevation may not correlate linearly with the severity of caries. Further studies with larger patient groups are needed to validate and expand upon these observations.

The primary limitation of this single-center study is the relatively small sample size. Additionally, anthropometric data such as height and weight were not recorded, which limited our ability to evaluate the potential confounding effects of body weight on inflammatory parameters. The absence of a statistically significant correlation between SII and the clinical caries indices (PUFA/pufa and DMFT/dmft) may also be influenced by this limitation, as nutritional status—including overweight and obesity—is known to affect systemic inflammation through adipose tissue-derived mediators.^27^ Furthermore, systemic inflammation may not always linearly reflect the severity of local oral disease, particularly in non-acute, chronic presentations. Individual variability in immune response or unmeasured confounding factors, such as stress or subclinical conditions, may have independently impacted inflammatory markers. Nevertheless, to the best of our knowledge, this study is among the limited number of investigations assessing systemic inflammation in children with untreated caries and their advanced complications.

## CONCLUSION

The present study data indicated that children with untreated caries may have changes in systemic inflammation markers. While our findings provide valuable insights, further studies with larger sample sizes and more detailed clinical and biological data—including anthropometric measurements—are needed to validate and expand upon our results. Meanwhile, preventive oral health programs should be developed and promoted to reduce the prevalence of caries and associated systemic health risks.

## REFERENCES 
